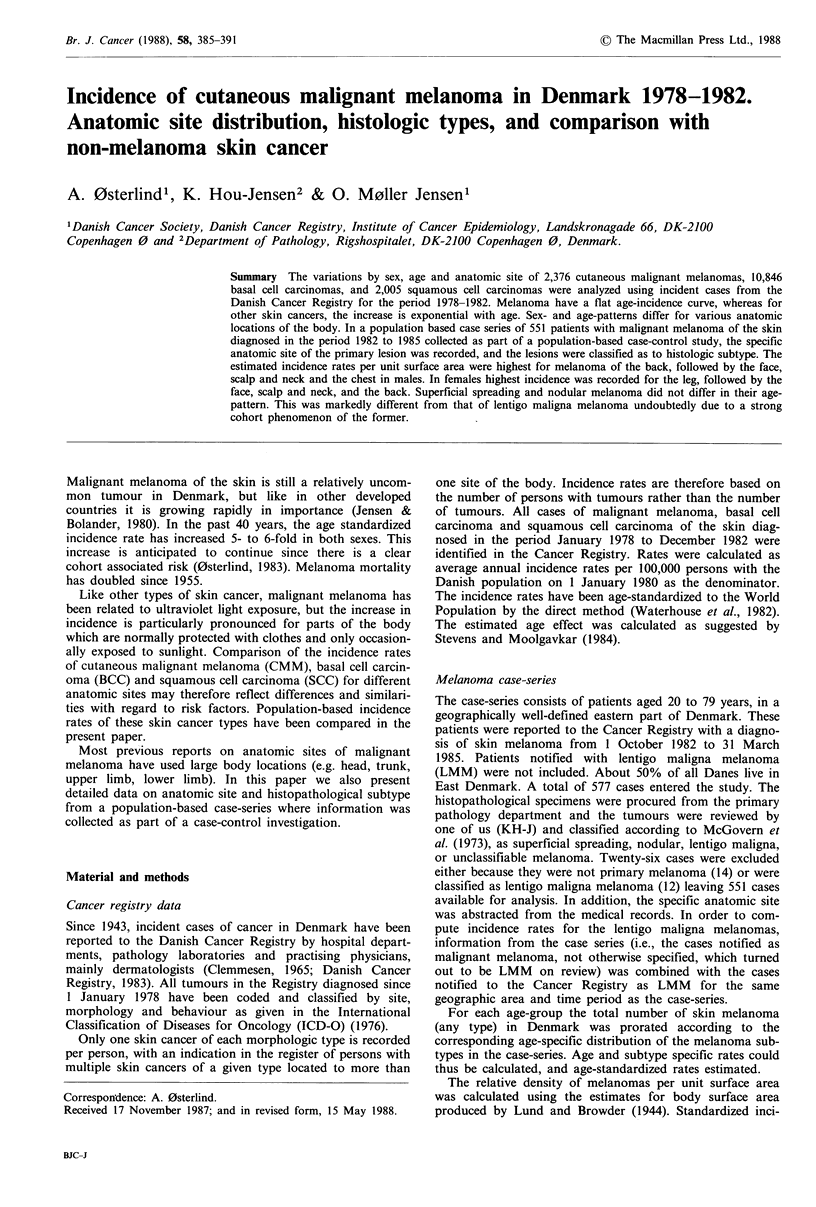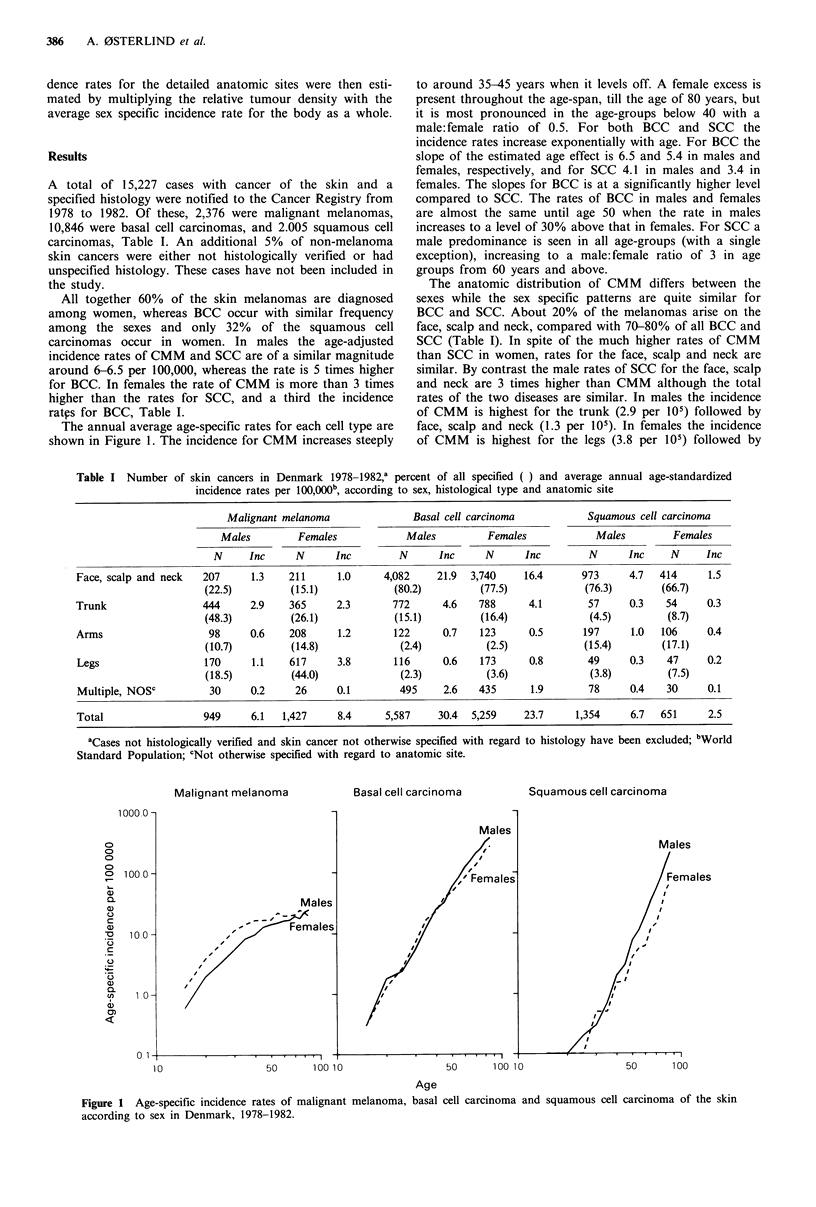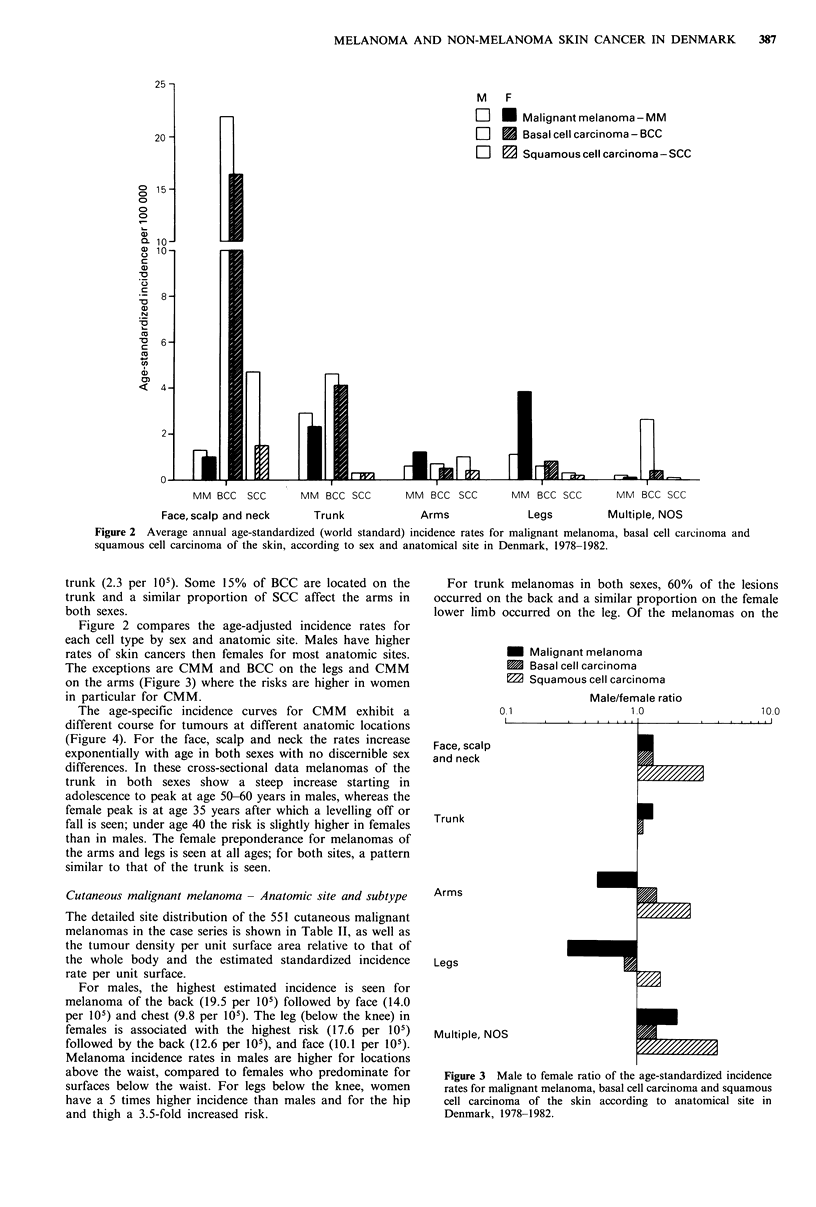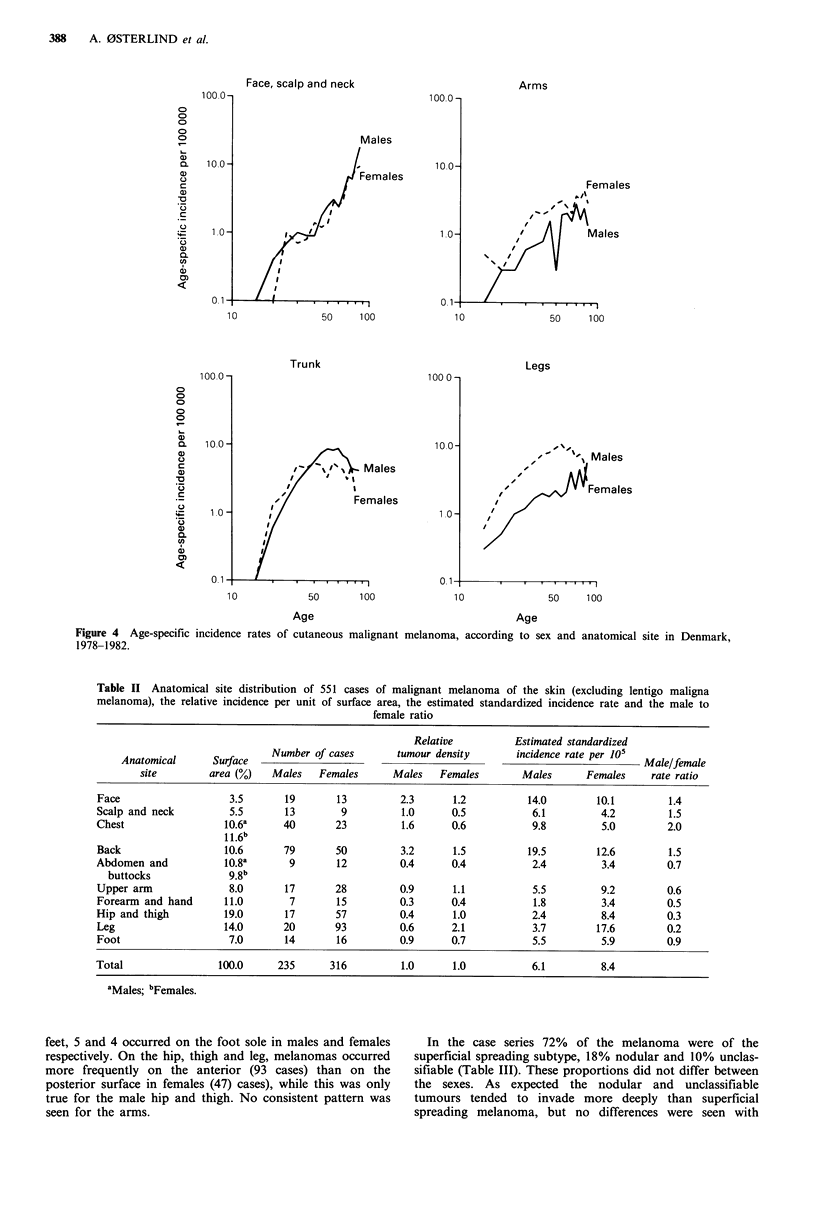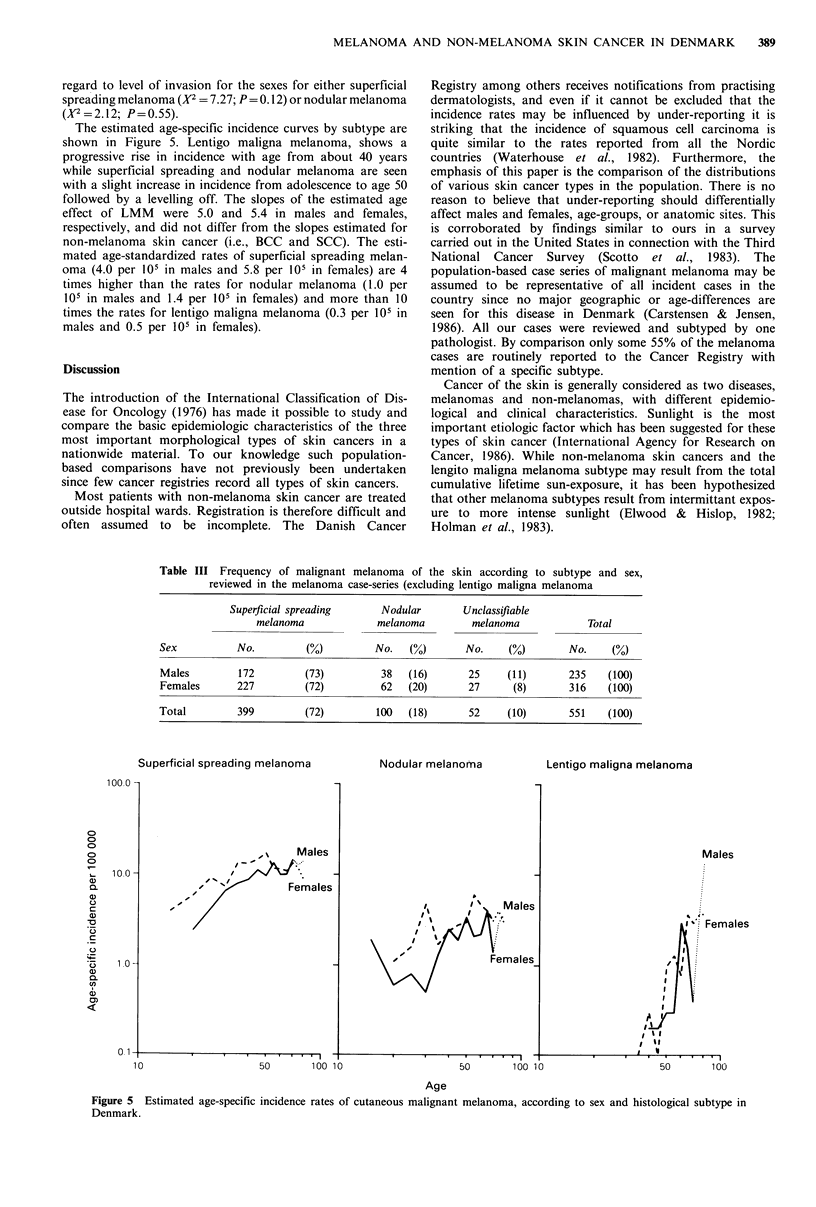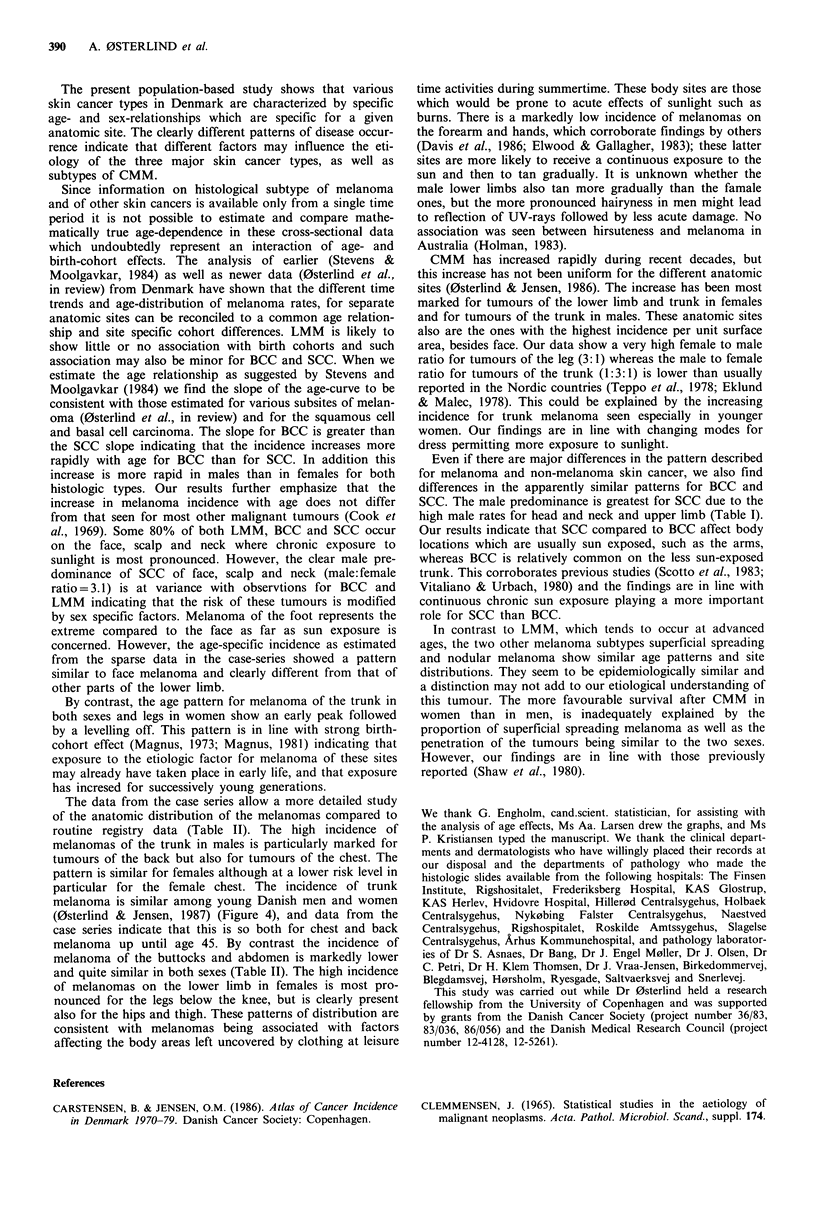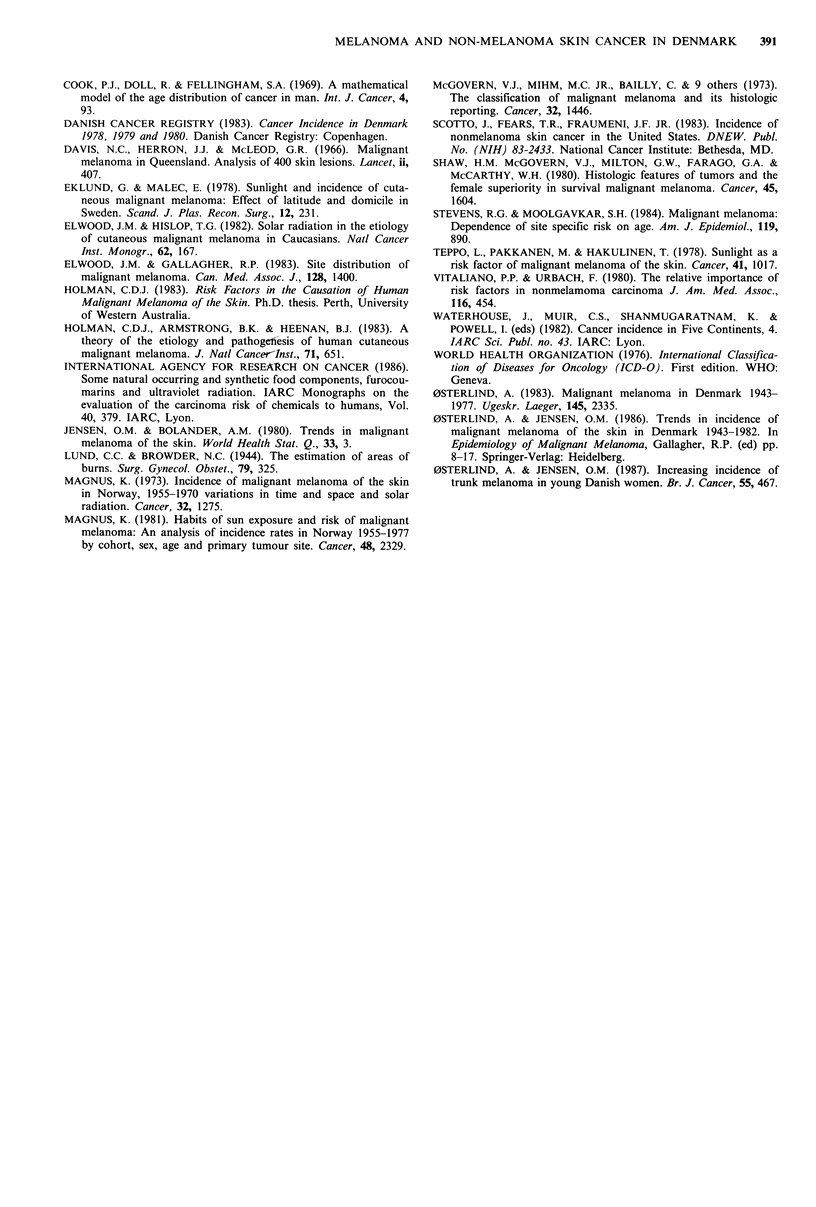# Incidence of cutaneous malignant melanoma in Denmark 1978-1982. Anatomic site distribution, histologic types, and comparison with non-melanoma skin cancer.

**DOI:** 10.1038/bjc.1988.225

**Published:** 1988-09

**Authors:** A. Osterlind, K. Hou-Jensen, O. MÃ¸ller Jensen

**Affiliations:** Danish Cancer Society, Institute of Cancer Epidemiology, Copenhagen.

## Abstract

The variations by sex, age and anatomic site of 2,376 cutaneous malignant melanomas, 10,846 basal cell carcinomas, and 2,005 squamous cell carcinomas were analyzed using incident cases from the Danish Cancer Registry for the period 1978-1982. Melanoma have a flat age-incidence curve, whereas for other skin cancers, the increase is exponential with age. Sex- and age-patterns differ for various anatomic locations of the body. In a population based case series of 551 patients with malignant melanoma of the skin diagnosed in the period 1982 to 1985 collected as part of a population-based case-control study, the specific anatomic site of the primary lesion was recorded, and the lesions were classified as to histologic subtype. The estimated incidence rates per unit surface area were highest for melanoma of the back, followed by the face, scalp and neck, and the chest in males. In females highest incidence was recorded for the leg, followed by the face, scalp and neck, and the back. Superficial spreading and nodular melanoma did not differ in their age-pattern. This was markedly different from that of lentigo maligna melanoma undoubtedly due to a strong cohort phenomenon of the former.


*Br. J. Cancer* (1988), **58**, 385–391 © The Macmillan Press Ltd., 1988

### Incidence of cutaneous malignant melanoma in Denmark 1978–1982. Anatomic site distribution, histologic types, and comparison with non-melanoma skin cancer

A. Østerlind[1], K. Hou-Jensen[2] & O. Møller Jensen[1]

[1]*Danish Cancer Society, Danish Cancer Registry, Institute of Cancer Epidemiology, Landskronagade 66, DK-2100 Copenhagen Ø and* [2]*Department of Pathology, Rigshospitalet, DK-2100 Copenhagen Ø, Denmark.*

**Summary** The variations by sex, age and anatomic site of 2,376 cutaneous malignant melanomas, 10,846 basal cell carcinomas, and 2,005 squamous cell carcinomas were analyzed using incident cases from the Danish Cancer Registry for the period 1978–1982. Melanoma have a flat age-incidence curve, whereas for other skin cancers, the increase is exponential with age. Sex- and age-patterns differ for various anatomic locations of the body. In a population based case series of 551 patients with malignant melanoma of the skin diagnosed in the period 1982 to 1985 collected as part of a population-based case-control study, the specific anatomic site of the primary lesion was recorded, and the lesions were classified as to histologic subtype. The estimated incidence rates per unit surface area were highest for melanoma of the back, followed by the face, scalp and neck and the chest in males. In females highest incidence was recorded for the leg, followed by the face, scalp and neck, and the back. Superficial spreading and nodular melanoma did not differ in their age-pattern. This was markedly different from that of lentigo maligna melanoma undoubtedly due to a strong cohort phenomenon of the former.

Malignant melanoma of the skin is still a relatively uncommon tumour in Denmark, but like in other developed countries it is growing rapidly in importance (Jensen & Bolander, 1980). In the past 40 years, the age standardized incidence rate has increased 5- to 6-fold in both sexes. This increase is anticipated to continue since there is a clear cohort associated risk (Østerlind, 1983). Melanoma mortality has doubled since 1955.

Like other types of skin cancer, malignant melanoma has been related to ultraviolet light exposure, but the increase in incidence is particularly pronounced for parts of the body which are normally protected with clothes and only occasionally exposed to sunlight. Comparison of the incidence rates of cutaneous malignant melanoma (CMM), basal cell carcinoma (BCC) and squamous cell carcinoma (SCC) for different anatomic sites may therefore reflect differences and similarities with regard to risk factors. Population-based incidence rates of these skin cancer types have been compared in the present paper.

Most previous reports on anatomic sites of malignant melanoma have used large body locations (e.g. head, trunk, upper limb, lower limb). In this paper we also present detailed data on anatomic site and histopathological subtype from a population-based case-series where information was collected as part of a case-control investigation.

### Material and methods

### *Cancer registry data*

Since 1943, incident cases of cancer in Denmark have been reported to the Danish Cancer Registry by hospital departments, pathology laboratories and practising physicians, mainly dermatologists (Clemmesen, 1965; Danish Cancer Registry, 1983). All tumours in the Registry diagnosed since 1 January 1978 have been coded and classified by site, morphology and behaviour as given in the International Classification of Diseases for Oncology (ICD-O) (1976).

Only one skin cancer of each morphologic type is recorded per person, with an indication in the register of persons with multiple skin cancers of a given type located to more than one site of the body. Incidence rates are therefore based on the number of persons with tumours rather than the number of tumours. All cases of malignant melanoma, basal cell carcinoma and squamous cell carcinoma of the skin diagnosed in the period January 1978 to December 1982 were identified in the Cancer Registry. Rates were calculated as average annual incidence rates per 100,000 persons with the Danish population on 1 January 1980 as the denominator. The incidence rates have been age-standardized to the World Population by the direct method (Waterhouse *et al.*, 1982). The estimated age effect was calculated as suggested by Stevens and Moolgavkar (1984).

### *Melanoma case-series*

The case-series consists of patients aged 20 to 79 years, in a geographically well-defined eastern part of Denmark. These patients were reported to the Cancer Registry with a diagnosis of skin melanoma from 1 October 1982 to 31 March 1985. Patients notified with lentigo maligna melanoma (LMM) were not included. About 50% of all Danes live in East Denmark. A total of 577 cases entered the study. The histopathological specimens were procured from the primary pathology department and the tumours were reviewed by one of us (KH-J) and classified according to McGovern *et al.* (1973), as superficial spreading, nodular, lentigo maligna, or unclassifiable melanoma. Twenty-six cases were excluded either because they were not primary melanoma (14) or were classified as lentigo maligna melanoma (12) leaving 551 cases available for analysis. In addition, the specific anatomic site was abstracted from the medical records. In order to compute incidence rates for the lentigo maligna melanomas, information from the case series (i.e., the cases notified as malignant melanoma, not otherwise specified, which turned out to be LMM on review) was combined with the cases notified to the Cancer Registry as LMM for the same geographic area and time period as the case-series.

For each age-group the total number of skin melanoma (any type) in Denmark was prorated according to the corresponding age-specific distribution of the melanoma subtypes in the case-series. Age and subtype specific rates could thus be calculated, and age-standardized rates estimated.

The relative density of melanomas per unit surface area was calculated using the estimates for body surface area produced by Lund and Browder (1944). Standardized inci-

Correspondence: A. Østerlind.

Received 17 November 1987; and in revised form, 15 May 1988.

dence rates for the detailed anatomic sites were then estimated by multiplying the relative tumour density with the average sex specific incidence rate for the body as a whole.

### Results

A total of 15,227 cases with cancer of the skin and a specified histology were notified to the Cancer Registry from 1978 to 1982. Of these, 2,376 were malignant melanomas, 10,846 were basal cell carcinomas, and 2.005 squamous cell carcinomas, Table I. An additional 5% of non-melanoma skin cancers were either not histologically verified or had unspecified histology. These cases have not been included in the study.

All together 60% of the skin melanomas are diagnosed among women, whereas BCC occur with similar frequency among the sexes and only 32% of the squamous cell carcinomas occur in women. In males the age-adjusted incidence rates of CMM and SCC are of a similar magnitude around 6–6.5 per 100,000, whereas the rate is 5 times higher for BCC. In females the rate of CMM is more than 3 times higher than the rates for SCC, and a third the incidence ratęs for BCC, Table I.

The annual average age-specific rates for each cell type are shown in Figure 1. The incidence for CMM increases steeply to around 35–45 years when it levels off. A female excess is present throughout the age-span, till the age of 80 years, but it is most pronounced in the age-groups below 40 with a male:female ratio of 0.5. For both BCC and SCC the incidence rates increase exponentially with age. For BCC the slope of the estimated age effect is 6.5 and 5.4 in males and females, respectively, and for SCC 4.1 in males and 3.4 in females. The slopes for BCC is at a significantly higher level compared to SCC. The rates of BCC in males and females are almost the same until age 50 when the rate in males increases to a level of 30% above that in females. For SCC a male predominance is seen in all age-groups (with a single exception), increasing to a male:female ratio of 3 in age groups from 60 years and above.

The anatomic distribution of CMM differs between the sexes while the sex specific patterns are quite similar for BCC and SCC. About 20% of the melanomas arise on the face, scalp and neck, compared with 70–80% of all BCC and SCC (Table I). In spite of the much higher rates of CMM than SCC in women, rates for the face, scalp and neck are similar. By contrast the male rates of SCC for the face, scalp and neck are 3 times higher than CMM although the total rates of the two diseases are similar. In males the incidence of CMM is highest for the trunk (2.9 per $10^5$) followed by face, scalp and neck (1.3 per $10^5$). In females the incidence of CMM is highest for the legs (3.8 per $10^5$) followed by

**Table I** Number of skin cancers in Denmark 1978–1982,[a] percent of all specified ( ) and average annual age-standardized incidence rates per 100,000[b], according to sex, histological type and anatomic site

| | *Malignant melanoma* | | | | *Basal cell carcinoma* | | | | *Squamous cell carcinoma* | | | |
|---|---|---|---|---|---|---|---|---|---|---|---|---|
| | *Males* | | *Females* | | *Males* | | *Females* | | *Males* | | *Females* | |
| | *N* | *Inc* | *N* | *Inc* | *N* | *Inc* | *N* | *Inc* | *N* | *Inc* | *N* | *Inc* |
| Face, scalp and neck | 207 (22.5) | 1.3 | 211 (15.1) | 1.0 | 4,082 (80.2) | 21.9 | 3,740 (77.5) | 16.4 | 973 (76.3) | 4.7 | 414 (66.7) | 1.5 |
| Trunk | 444 (48.3) | 2.9 | 365 (26.1) | 2.3 | 772 (15.1) | 4.6 | 788 (16.4) | 4.1 | 57 (4.5) | 0.3 | 54 (8.7) | 0.3 |
| Arms | 98 (10.7) | 0.6 | 208 (14.8) | 1.2 | 122 (2.4) | 0.7 | 123 (2.5) | 0.5 | 197 (15.4) | 1.0 | 106 (17.1) | 0.4 |
| Legs | 170 (18.5) | 1.1 | 617 (44.0) | 3.8 | 116 (2.3) | 0.6 | 173 (3.6) | 0.8 | 49 (3.8) | 0.3 | 47 (7.5) | 0.2 |
| Multiple, NOS[c] | 30 | 0.2 | 26 | 0.1 | 495 | 2.6 | 435 | 1.9 | 78 | 0.4 | 30 | 0.1 |
| Total | 949 | 6.1 | 1,427 | 8.4 | 5,587 | 30.4 | 5,259 | 23.7 | 1,354 | 6.7 | 651 | 2.5 |

[a]Cases not histologically verified and skin cancer not otherwise specified with regard to histology have been excluded; [b]World Standard Population; [c]Not otherwise specified with regard to anatomic site.

**Figure 1** Age-specific incidence rates of malignant melanoma, basal cell carcinoma and squamous cell carcinoma of the skin according to sex in Denmark, 1978–1982.

**Figure 2** Average annual age-standardized (world standard) incidence rates for malignant melanoma, basal cell carcinoma and squamous cell carcinoma of the skin, according to sex and anatomical site in Denmark, 1978–1982.

trunk (2.3 per $10^5$). Some 15% of BCC are located on the trunk and a similar proportion of SCC affect the arms in both sexes.

Figure 2 compares the age-adjusted incidence rates for each cell type by sex and anatomic site. Males have higher rates of skin cancers then females for most anatomic sites. The exceptions are CMM and BCC on the legs and CMM on the arms (Figure 3) where the risks are higher in women in particular for CMM.

The age-specific incidence curves for CMM exhibit a different course for tumours at different anatomic locations (Figure 4). For the face, scalp and neck the rates increase exponentially with age in both sexes with no discernible sex differences. In these cross-sectional data melanomas of the trunk in both sexes show a steep increase starting in adolescence to peak at age 50–60 years in males, whereas the female peak is at age 35 years after which a levelling off or fall is seen; under age 40 the risk is slightly higher in females than in males. The female preponderance for melanomas of the arms and legs is seen at all ages; for both sites, a pattern similar to that of the trunk is seen.

*Cutaneous malignant melanoma – Anatomic site and subtype*

The detailed site distribution of the 551 cutaneous malignant melanomas in the case series is shown in Table II, as well as the tumour density per unit surface area relative to that of the whole body and the estimated standardized incidence rate per unit surface.

For males, the highest estimated incidence is seen for melanoma of the back (19.5 per $10^5$) followed by face (14.0 per $10^5$) and chest (9.8 per $10^5$). The leg (below the knee) in females is associated with the highest risk (17.6 per $10^5$) followed by the back (12.6 per $10^5$), and face (10.1 per $10^5$). Melanoma incidence rates in males are higher for locations above the waist, compared to females who predominate for surfaces below the waist. For legs below the knee, women have a 5 times higher incidence than males and for the hip and thigh a 3.5-fold increased risk.

For trunk melanomas in both sexes, 60% of the lesions occurred on the back and a similar proportion on the female lower limb occurred on the leg. Of the melanomas on the

**Figure 3** Male to female ratio of the age-standardized incidence rates for malignant melanoma, basal cell carcinoma and squamous cell carcinoma of the skin according to anatomical site in Denmark, 1978–1982.

**Figure 4** Age-specific incidence rates of cutaneous malignant melanoma, according to sex and anatomical site in Denmark, 1978–1982.

**Table II** Anatomical site distribution of 551 cases of malignant melanoma of the skin (excluding lentigo maligna melanoma), the relative incidence per unit of surface area, the estimated standardized incidence rate and the male to female ratio

| Anatomical site | Surface area (%) | Number of cases | | Relative tumour density | | Estimated standardized incidence rate per $10^5$ | | Male/female rate ratio |
|---|---|---|---|---|---|---|---|---|
| | | Males | Females | Males | Females | Males | Females | |
| Face | 3.5 | 19 | 13 | 2.3 | 1.2 | 14.0 | 10.1 | 1.4 |
| Scalp and neck | 5.5 | 13 | 9 | 1.0 | 0.5 | 6.1 | 4.2 | 1.5 |
| Chest | 10.6[a] 11.6[b] | 40 | 23 | 1.6 | 0.6 | 9.8 | 5.0 | 2.0 |
| Back | 10.6 | 79 | 50 | 3.2 | 1.5 | 19.5 | 12.6 | 1.5 |
| Abdomen and buttocks | 10.8[a] 9.8[b] | 9 | 12 | 0.4 | 0.4 | 2.4 | 3.4 | 0.7 |
| Upper arm | 8.0 | 17 | 28 | 0.9 | 1.1 | 5.5 | 9.2 | 0.6 |
| Forearm and hand | 11.0 | 7 | 15 | 0.3 | 0.4 | 1.8 | 3.4 | 0.5 |
| Hip and thigh | 19.0 | 17 | 57 | 0.4 | 1.0 | 2.4 | 8.4 | 0.3 |
| Leg | 14.0 | 20 | 93 | 0.6 | 2.1 | 3.7 | 17.6 | 0.2 |
| Foot | 7.0 | 14 | 16 | 0.9 | 0.7 | 5.5 | 5.9 | 0.9 |
| Total | 100.0 | 235 | 316 | 1.0 | 1.0 | 6.1 | 8.4 | |

[a]Males; [b]Females.

feet, 5 and 4 occurred on the foot sole in males and females respectively. On the hip, thigh and leg, melanomas occurred more frequently on the anterior (93 cases) than on the posterior surface in females (47) cases), while this was only true for the male hip and thigh. No consistent pattern was seen for the arms.

In the case series 72% of the melanoma were of the superficial spreading subtype, 18% nodular and 10% unclassifiable (Table III). These proportions did not differ between the sexes. As expected the nodular and unclassifiable tumours tended to invade more deeply than superficial spreading melanoma, but no differences were seen with regard to level of invasion for the sexes for either superficial spreading melanoma ($X^2=7.27$; $P=0.12$) or nodular melanoma ($X^2=2.12$; $P=0.55$).

The estimated age-specific incidence curves by subtype are shown in Figure 5. Lentigo maligna melanoma, shows a progressive rise in incidence with age from about 40 years while superficial spreading and nodular melanoma are seen with a slight increase in incidence from adolescence to age 50 followed by a levelling off. The slopes of the estimated age effect of LMM were 5.0 and 5.4 in males and females, respectively, and did not differ from the slopes estimated for non-melanoma skin cancer (i.e., BCC and SCC). The estimated age-standardized rates of superficial spreading melanoma (4.0 per $10^5$ in males and 5.8 per $10^5$ in females) are 4 times higher than the rates for nodular melanoma (1.0 per $10^5$ in males and 1.4 per $10^5$ in females) and more than 10 times the rates for lentigo maligna melanoma (0.3 per $10^5$ in males and 0.5 per $10^5$ in females).

### Discussion

The introduction of the International Classification of Disease for Oncology (1976) has made it possible to study and compare the basic epidemiologic characteristics of the three most important morphological types of skin cancers in a nationwide material. To our knowledge such population-based comparisons have not previously been undertaken since few cancer registries record all types of skin cancers.

Most patients with non-melanoma skin cancer are treated outside hospital wards. Registration is therefore difficult and often assumed to be incomplete. The Danish Cancer Registry among others receives notifications from practising dermatologists, and even if it cannot be excluded that the incidence rates may be influenced by under-reporting it is striking that the incidence of squamous cell carcinoma is quite similar to the rates reported from all the Nordic countries (Waterhouse *et al.*, 1982). Furthermore, the emphasis of this paper is the comparison of the distributions of various skin cancer types in the population. There is no reason to believe that under-reporting should differentially affect males and females, age-groups, or anatomic sites. This is corroborated by findings similar to ours in a survey carried out in the United States in connection with the Third National Cancer Survey (Scotto *et al.*, 1983). The population-based case series of malignant melanoma may be assumed to be representative of all incident cases in the country since no major geographic or age-differences are seen for this disease in Denmark (Carstensen & Jensen, 1986). All our cases were reviewed and subtyped by one pathologist. By comparison only some 55% of the melanoma cases are routinely reported to the Cancer Registry with mention of a specific subtype.

Cancer of the skin is generally considered as two diseases, melanomas and non-melanomas, with different epidemiological and clinical characteristics. Sunlight is the most important etiologic factor which has been suggested for these types of skin cancer (International Agency for Research on Cancer, 1986). While non-melanoma skin cancers and the lengito maligna melanoma subtype may result from the total cumulative lifetime sun-exposure, it has been hypothesized that other melanoma subtypes result from intermittant exposure to more intense sunlight (Elwood & Hislop, 1982; Holman *et al.*, 1983).

**Table III** Frequency of malignant melanoma of the skin according to subtype and sex, reviewed in the melanoma case-series (excluding lentigo maligna melanoma

| | *Superficial spreading melanoma* | | *Nodular melanoma* | | *Unclassifiable melanoma* | | *Total* | |
|---|---|---|---|---|---|---|---|---|
| *Sex* | *No.* | *(%)* | *No.* | *(%)* | *No.* | *(%)* | *No.* | *(%)* |
| Males | 172 | (73) | 38 | (16) | 25 | (11) | 235 | (100) |
| Females | 227 | (72) | 62 | (20) | 27 | (8) | 316 | (100) |
| Total | 399 | (72) | 100 | (18) | 52 | (10) | 551 | (100) |

**Figure 5** Estimated age-specific incidence rates of cutaneous malignant melanoma, according to sex and histological subtype in Denmark.

The present population-based study shows that various skin cancer types in Denmark are characterized by specific age- and sex-relationships which are specific for a given anatomic site. The clearly different patterns of disease occurrence indicate that different factors may influence the etiology of the three major skin cancer types, as well as subtypes of CMM.

Since information on histological subtype of melanoma and of other skin cancers is available only from a single time period it is not possible to estimate and compare mathematically true age-dependence in these cross-sectional data which undoubtedly represent an interaction of age- and birth-cohort effects. The analysis of earlier (Stevens & Moolgavkar, 1984) as well as newer data (Østerlind *et al.*, in review) from Denmark have shown that the different time trends and age-distribution of melanoma rates, for separate anatomic sites can be reconciled to a common age relationship and site specific cohort differences. LMM is likely to show little or no association with birth cohorts and such association may also be minor for BCC and SCC. When we estimate the age relationship as suggested by Stevens and Moolgavkar (1984) we find the slope of the age-curve to be consistent with those estimated for various subsites of melanoma (Østerlind *et al.*, in review) and for the squamous cell and basal cell carcinoma. The slope for BCC is greater than the SCC slope indicating that the incidence increases more rapidly with age for BCC than for SCC. In addition this increase is more rapid in males than in females for both histologic types. Our results further emphasize that the increase in melanoma incidence with age does not differ from that seen for most other malignant tumours (Cook *et al.*, 1969). Some 80% of both LMM, BCC and SCC occur on the face, scalp and neck where chronic exposure to sunlight is most pronounced. However, the clear male predominance of SCC of face, scalp and neck (male:female ratio = 3.1) is at variance with observtions for BCC and LMM indicating that the risk of these tumours is modified by sex specific factors. Melanoma of the foot represents the extreme compared to the face as far as sun exposure is concerned. However, the age-specific incidence as estimated from the sparse data in the case-series showed a pattern similar to face melanoma and clearly different from that of other parts of the lower limb.

By contrast, the age pattern for melanoma of the trunk in both sexes and legs in women show an early peak followed by a levelling off. This pattern is in line with strong birth-cohort effect (Magnus, 1973; Magnus, 1981) indicating that exposure to the etiologic factor for melanoma of these sites may already have taken place in early life, and that exposure has incresed for successively young generations.

The data from the case series allow a more detailed study of the anatomic distribution of the melanomas compared to routine registry data (Table II). The high incidence of melanomas of the trunk in males is particularly marked for tumours of the back but also for tumours of the chest. The pattern is similar for females although at a lower risk level in particular for the female chest. The incidence of trunk melanoma is similar among young Danish men and women (Østerlind & Jensen, 1987) (Figure 4), and data from the case series indicate that this is so both for chest and back melanoma up until age 45. By contrast the incidence of melanoma of the buttocks and abdomen is markedly lower and quite similar in both sexes (Table II). The high incidence of melanomas on the lower limb in females is most pronounced for the legs below the knee, but is clearly present also for the hips and thigh. These patterns of distribution are consistent with melanomas being associated with factors affecting the body areas left uncovered by clothing at leisure time activities during summertime. These body sites are those which would be prone to acute effects of sunlight such as burns. There is a markedly low incidence of melanomas on the forearm and hands, which corroborate findings by others (Davis *et al.*, 1986; Elwood & Gallagher, 1983); these latter sites are more likely to receive a continuous exposure to the sun and then to tan gradually. It is unknown whether the male lower limbs also tan more gradually than the famale ones, but the more pronounced hairyness in men might lead to reflection of UV-rays followed by less acute damage. No association was seen between hirsuteness and melanoma in Australia (Holman, 1983).

CMM has increased rapidly during recent decades, but this increase has not been uniform for the different anatomic sites (Østerlind & Jensen, 1986). The increase has been most marked for tumours of the lower limb and trunk in females and for tumours of the trunk in males. These anatomic sites also are the ones with the highest incidence per unit surface area, besides face. Our data show a very high female to male ratio for tumours of the leg (3:1) whereas the male to female ratio for tumours of the trunk (1:3:1) is lower than usually reported in the Nordic countries (Teppo *et al.*, 1978; Eklund & Malec, 1978). This could be explained by the increasing incidence for trunk melanoma seen especially in younger women. Our findings are in line with changing modes for dress permitting more exposure to sunlight.

Even if there are major differences in the pattern described for melanoma and non-melanoma skin cancer, we also find differences in the apparently similar patterns for BCC and SCC. The male predominance is greatest for SCC due to the high male rates for head and neck and upper limb (Table I). Our results indicate that SCC compared to BCC affect body locations which are usually sun exposed, such as the arms, whereas BCC is relatively common on the less sun-exposed trunk. This corroborates previous studies (Scotto *et al.*, 1983; Vitaliano & Urbach, 1980) and the findings are in line with continuous chronic sun exposure playing a more important role for SCC than BCC.

In contrast to LMM, which tends to occur at advanced ages, the two other melanoma subtypes superficial spreading and nodular melanoma show similar age patterns and site distributions. They seem to be epidemiologically similar and a distinction may not add to our etiological understanding of this tumour. The more favourable survival after CMM in women than in men, is inadequately explained by the proportion of superficial spreading melanoma as well as the penetration of the tumours being similar to the two sexes. However, our findings are in line with those previously reported (Shaw *et al.*, 1980).

We thank G. Engholm, cand.scient. statistician, for assisting with the analysis of age effects, Ms Aa. Larsen drew the graphs, and Ms P. Kristiansen typed the manuscript. We thank the clinical departments and dermatologists who have willingly placed their records at our disposal and the departments of pathology who made the histologic slides available from the following hospitals: The Finsen Institute, Rigshositalet, Frederiksberg Hospital, KAS Glostrup, KAS Herlev, Hvidovre Hospital, Hillerød Centralsygehus, Holbaek Centralsygehus, Nykøbing Falster Centralsygehus, Naestved Centralsygehus, Rigshospitalet, Roskilde Amtssygehus, Slagelse Centralsygehus, Århus Kommunehospital, and pathology laboratories of Dr S. Asnaes, Dr Bang, Dr J. Engel Møller, Dr J. Olsen, Dr C. Petri, Dr H. Klem Thomsen, Dr J. Vraa-Jensen, Birkedommervej, Blegdamsvej, Hørsholm, Ryesgade, Saltvaerksvej and Snerlevej.

This study was carried out while Dr Østerlind held a research fellowship from the University of Copenhagen and was supported by grants from the Danish Cancer Society (project number 36/83, 83/036, 86/056) and the Danish Medical Research Council (project number 12-4128, 12-5261).

### References

CARSTENSEN, B. & JENSEN, O.M. (1986). *Atlas of Cancer Incidence in Denmark 1970–79*. Danish Cancer Society: Copenhagen.

CLEMMENSEN, J. (1965). Statistical studies in the aetiology of malignant neoplasms. *Acta. Pathol. Microbiol. Scand.*, suppl. **174**.

COOK, P.J., DOLL, R. & FELLINGHAM, S.A. (1969). A mathematical model of the age distribution of cancer in man. *Int. J. Cancer*, **4**, 93.

DANISH CANCER REGISTRY (1983). *Cancer Incidence in Denmark 1978, 1979 and 1980*. Danish Cancer Registry: Copenhagen.

DAVIS, N.C., HERRON, J.J. & McLEOD, G.R. (1966). Malignant melanoma in Queensland. Analysis of 400 skin lesions. *Lancet*, **ii**, 407.

EKLUND, G. & MALEC, E. (1978). Sunlight and incidence of cutaneous malignant melanoma: Effect of latitude and domicile in Sweden. *Scand. J. Plas. Recon. Surg.*, **12**, 231.

ELWOOD, J.M. & HISLOP, T.G. (1982). Solar radiation in the etiology of cutaneous malignant melanoma in Caucasians. *Natl Cancer Inst. Monogr.*, **62**, 167.

ELWOOD, J.M. & GALLAGHER, R.P. (1983). Site distribution of malignant melanoma. *Can. Med. Assoc. J.*, **128**, 1400.

HOLMAN, C.D.J. (1983). *Risk Factors in the Causation of Human Malignant Melanoma of the Skin*. Ph.D. thesis. Perth, University of Western Australia.

HOLMAN, C.D.J., ARMSTRONG, B.K. & HEENAN, B.J. (1983). A theory of the etiology and pathogenesis of human cutaneous malignant melanoma. *J. Natl Cancer Inst.*, **71**, 651.

INTERNATIONAL AGENCY FOR RESEARCH ON CANCER (1986). Some natural occurring and synthetic food components, furocoumarins and ultraviolet radiation. IARC Monographs on the evaluation of the carcinoma risk of chemicals to humans, Vol. 40, 379. IARC, Lyon.

JENSEN, O.M. & BOLANDER, A.M. (1980). Trends in malignant melanoma of the skin. *World Health Stat. Q.*, **33**, 3.

LUND, C.C. & BROWDER, N.C. (1944). The estimation of areas of burns. *Surg. Gynecol. Obstet.*, **79**, 325.

MAGNUS, K. (1973). Incidence of malignant melanoma of the skin in Norway, 1955–1970 variations in time and space and solar radiation. *Cancer*, **32**, 1275.

MAGNUS, K. (1981). Habits of sun exposure and risk of malignant melanoma: An analysis of incidence rates in Norway 1955–1977 by cohort, sex, age and primary tumour site. *Cancer*, **48**, 2329.

McGOVERN, V.J., MIHM, M.C. JR., BAILLY, C. & 9 others (1973). The classification of malignant melanoma and its histologic reporting. *Cancer*, **32**, 1446.

SCOTTO, J., FEARS, T.R., FRAUMENI, J.F. JR. (1983). Incidence of nonmelanoma skin cancer in the United States. *DNEW. Publ. No. (NIH) 83-2433*. National Cancer Institute: Bethesda, MD.

SHAW, H.M. McGOVERN, V.J., MILTON, G.W., FARAGO, G.A. & McCARTHY, W.H. (1980). Histologic features of tumors and the female superiority in survival malignant melanoma. *Cancer*, **45**, 1604.

STEVENS, R.G. & MOOLGAVKAR, S.H. (1984). Malignant melanoma: Dependence of site specific risk on age. *Am. J. Epidemiol.*, **119**, 890.

TEPPO, L., PAKKANEN, M. & HAKULINEN, T. (1978). Sunlight as a risk factor of malignant melanoma of the skin. *Cancer*, **41**, 1017.

VITALIANO, P.P. & URBACH, F. (1980). The relative importance of risk factors in nonmelamoma carcinoma *J. Am. Med. Assoc.*, **116**, 454.

WATERHOUSE, J., MUIR, C.S., SHANMUGARATNAM, K. & POWELL, I. (eds) (1982). Cancer incidence in Five Continents, 4. *IARC Sci. Publ. no. 43*. IARC: Lyon.

WORLD HEALTH ORGANIZATION (1976). *International Classification of Diseases for Oncology (ICD-O)*. First edition. WHO: Geneva.

ØSTERLIND, A. (1983). Malignant melanoma in Denmark 1943–1977. *Ugeskr. Laeger*, **145**, 2335.

ØSTERLIND, A. & JENSEN, O.M. (1986). Trends in incidence of malignant melanoma of the skin in Denmark 1943–1982. In *Epidemiology of Malignant Melanoma*, Gallagher, R.P. (ed) pp. 8–17. Springer-Verlag: Heidelberg.

ØSTERLIND, A. & JENSEN, O.M. (1987). Increasing incidence of trunk melanoma in young Danish women. *Br. J. Cancer*, **55**, 467.